# Seasonal changes in lipid profiles and homocysteine in patients with and without coronary heart disease: a real-world paired retrospective study in a cold-climate region

**DOI:** 10.3389/fcvm.2026.1774825

**Published:** 2026-03-25

**Authors:** Tao Liu, Lin Xia, Yingjie Zhang, Entao Zhou, Huishan Wang

**Affiliations:** 1 The First Affiliated Hospital of Dalian Medical University, Dalian Medical University, Dalian, China; 2 State Key Laboratory of Frigid Zone Cardiovascular Disease, Department of Cardiovascular Surgery, General Hospital of Northern Theater Command, Shenyang, China

**Keywords:** coronary heart disease, homocysteine, lipids, real-world data, seasonality

## Abstract

**Background:**

Seasonal variation in cardiovascular events is well recognized in cold-climate regions. However, whether lipid profiles and homocysteine exhibit consistent seasonal patterns, and whether these patterns differ between individuals with and without coronary heart disease (CHD), remains incompletely characterized in real-world clinical settings.

**Methods:**

We conducted a retrospective, paired study including patients who underwent first-time measurements of lipid profiles and homocysteine in both summer (June–August) and winter (December–February) at a single center over a three-year period. Participants were stratified by CHD status. Within-individual seasonal differences were assessed using paired statistical tests. Seasonal changes were quantified as Δ (Winter − Summer), and between-group differences in Δ were compared. Effect sizes and complementary visualizations were used to characterize the magnitude and heterogeneity of seasonal responses.

**Results:**

Seasonal differences in lipid parameters and homocysteine were detectable but modest. Individuals without CHD showed greater seasonal responsiveness, with winter increases in LDL-C and reductions in total cholesterol, triglycerides, and homocysteine. In contrast, CHD patients exhibited attenuated seasonal variation, with a mild winter increase in LDL-C and a tendency toward lower HDL-C. Between-group comparisons of Δ revealed significant heterogeneity for triglycerides (*p* < 0.001) and HDL-C (*p* = 0.036), indicating discordant seasonal response patterns. Effect size analyses consistently suggested small effects across biomarkers.

**Conclusions:**

Seasonal variation in lipid profiles and homocysteine is modest but heterogeneous. Non-CHD individuals show greater seasonal sensitivity, whereas CHD patients exhibit blunted or directionally altered responses. These findings highlight the importance of incorporating seasonal context into lipid interpretation and individualized cardiovascular risk management in cold-climate regions.

## Introduction

Coronary heart disease (CHD) remains a leading contributor to global morbidity and mortality. Beyond traditional risk factors, environmental exposures—especially ambient temperature—have increasingly been recognized as potential triggers for acute cardiovascular events. Large population-based analyses have demonstrated that colder temperatures are associated with higher short-term risk of myocardial infarction, providing a plausible epidemiological basis for winter excess in cardiovascular outcomes (1–3). While acute hemodynamic and neurohumoral responses to cold (e.g., sympathetic activation, vasoconstriction, and blood pressure elevation) are often invoked, the extent to which cold seasons shape chronic or intermediate cardiometabolic phenotypes—such as lipid profiles and homocysteine—remains variably reported and may differ across populations.

Seasonal variability in serum lipids has been documented for decades, with several studies reporting higher cholesterol levels during winter compared with summer. Importantly, the clinical interpretation of lipid measurements may be confounded by season-of-sampling, and the amplitude of seasonal variability may be influenced by baseline cardiometabolic risk, plasma volume shifts, physical activity, and other behavioral factors (4, 5). For patients with established atherosclerotic cardiovascular disease, contemporary guidelines emphasize intensive lipoprotein cholesterol (LDL-C) lowering and comprehensive lipid management, largely driven by pharmacotherapy (e.g., statins, ezetimibe, PCSK9 inhibitors) and lifestyle interventions (6, 7). It is therefore plausible that medication use and structured secondary prevention could “flatten” seasonal lipid oscillations in CHD patients, producing a more attenuated seasonal signal compared with non-CHD individuals.

Mechanistic insights from experimental studies further suggest that cold exposure may modulate lipid metabolism and atherosclerosis progression. In atherosclerosis-prone mouse models, cold exposure has been linked to accelerated lesion development through cold-triggered lipolysis and increased circulating atherogenic lipoprotein remnants; such effects may be tied to thermogenic pathways (8). These mechanistic observations, together with epidemiological evidence on winter cardiovascular risk, motivate the need for real-world paired investigations that compare within-individual winter–summer changes and quantify whether CHD status modifies seasonal metabolic responses.

In this context, we leveraged real-world clinical laboratory data from a cold-climate region and adopted a paired design—using each individual as their own control—to reduce between-person confounding. We aimed to (i) characterize within-individual seasonal differences in LDL-C, high-density lipoprotein cholesterol (HDL-C), total cholesterol (TC), triglycerides (TG), and homocysteine (Hcy); (ii) compare seasonal response magnitudes between CHD and non-CHD groups using Δ (Winter − Summer); and (iii) quantify effect sizes and assess robustness to distributional outliers. By focusing on both statistical detectability and effect size, we sought to provide an editor- and reviewer-friendly interpretation that distinguishes mild seasonal shifts from clinically actionable patterns.

## Methods

### Study design and data source

This study was designed as a real-world, retrospective, paired observational analysis. Clinical laboratory data were extracted from the electronic hospital information system of the General Hospital of Northern Theater Command, Shenyang, a tertiary referral center located in Northeast China, a cold-climate region with a high volume of cardiovascular patients, covering a multi-year period from 2022 to 2024. The paired design enabled within-individual comparisons between summer and winter measurements, thereby minimizing confounding from stable individual-level characteristics, including sex, genetic background, and long-term lifestyle patterns.

The study protocol was conducted in accordance with the principles of the Declaration of Helsinki. Given the retrospective nature of anonymized data extraction and analysis, the requirement for informed consent was waived by the institutional ethics committee, in line with local regulatory standards.

### Study population

Eligible participants were adults who met all of the following criteria: (1) Had at least one first-time measurement of serum lipids and/or Hcy during the summer season (defined as June–August); (2) Had at least one first-time measurement of the same biomarker during the winter season (defined as December–February); (3) Both measurements were available within the study period, enabling within-individual seasonal pairing.

To ensure comparability, only the first available measuremen**t** in each season was retained for analysis. Individuals with documented acute myocardial infarction at the time of laboratory testing were excluded. Participants were classified into two clinical strata based on diagnostic records: (1) CHD group; (2) Non-CHD group.

Baseline variables were extracted from the electronic hospital information system, including age, sex, body mass index (BMI), major cardiovascular risk factors (hypertension, diabetes mellitus, and smoking status), and selected comorbidities documented in diagnostic fields (e.g., chronic kidney disease and prior stroke/TIA). Medication use at the time of testing—particularly lipid-lowering therapy (overall and by class: statins, ezetimibe, and PCSK9 inhibitors), as well as folate/B-vitamin supplementation—was also captured given its marked imbalance between participants with and without CHD and its potential to modify seasonal biomarker variation. These variables were used to characterize the cohort and to inform sensitivity and stratified analyses evaluating whether baseline risk profiles and LLT status influenced within-individual seasonal changes in lipid and homocysteine levels.

### Biomarkers and laboratory measurements

The biomarkers of interest included: (1) LDL-C; (2) HDL-C, (3) TC; (4) TG; (5) Hcy. All measurements were performed by the hospital's central laboratory using standardized, quality-controlled procedures consistent with routine clinical practice. Units were reported as mmol/L for lipid parameters and μmol/L for Hcy.

### Medication ascertainment

Lipid-lowering therapy (LLT) status was ascertained from structured medication records and prescription fields in the hospital information system, capturing any LLT use and, when available, specific classes (statins, ezetimibe, and PCSK9 inhibitors). Participants were classified as receiving LLT if an active prescription was recorded within a prespecified window surrounding the date of each laboratory test. Where feasible, “stable LLT” was defined as concordant LLT status across the paired summer and winter measurement windows to minimize exposure misclassification. Given the substantial imbalance in LLT use between CHD and non-CHD participants at baseline, we performed prespecified stratified and sensitivity analyses by LLT status to evaluate whether treatment modified within-individual seasonal changes in lipids and homocysteine. These analyses compared seasonal differences (Δ = winter − summer) between CHD and non-CHD participants within LLT strata, and were interpreted alongside the overall within-group seasonal comparisons and between-group contrasts.

### Definition of seasonal difference

For each individual and each biomarker, the seasonal difference was defined as:

Δ=Wintervalue−Summervalue

A positive Δ indicated a higher level in winter compared with summer, whereas a negative Δ indicated a lower winter level. This definition was applied uniformly across biomarkers and analyses.

### Statistical analysis

All statistical analyses were performed using R (version ≥4.2.0). Analyses followed a predefined workflow emphasizing transparency and reproducibility. Continuous variables were summarized as mean ± standard deviation (SD). For completeness and assessment of distributional properties, medians and interquartile ranges (IQRs) were also computed, although mean ± SD was used as the primary reporting format in tables. Within each CHD stratum, paired comparisons between summer and winter measurements were conducted separately for each biomarker. If the distribution of Δ approximated normality (assessed using the Shapiro–Wilk test, with flexibility for large sample sizes), paired *t*-tests were applied. If Δ showed marked skewness or non-normality, Wilcoxon signed-rank tests were used. To assess whether seasonal responses differed between CHD and non-CHD groups, Δ values were compared between groups for each biomarker. Independent-sample *t*-tests were used as the default approach. When Δ distributions were clearly non-normal, Wilcoxon rank-sum tests were considered. To quantify the magnitude of seasonal effects beyond statistical significance, effect sizes were calculated using Cohen's *d*, based on Δ values within each CHD stratum. To evaluate robustness to extreme values, sensitivity analyses were performed by winsorizing Δ values at the 1st and 99th percentiles. Key between-group comparisons were re-estimated and visualized. Two-sided *p* values <0.05 were considered statistically significant. Given the exploratory nature of this real-world analysis and the emphasis on effect sizes and pattern consistency, formal adjustment for multiple comparisons was not applied in the primary analysis.

## Results

### Baseline characteristics

Baseline demographic and clinical characteristics stratified by CHD status are summarized in Table 1. Compared with participants without CHD, those with CHD were older, more often male, and had a higher burden of cardiometabolic risk factors and comorbidities, including hypertension, diabetes mellitus, smoking history, chronic kidney disease, and prior stroke/TIA (Table 1). These between-group differences align with the clinical profile of established CHD and provide important context for interpreting heterogeneity in seasonal biomarker responses.

**Table 1:** Baseline characteristics of participants by CHD status.

Characteristic	Non-CHD (*n* = 1,812)	CHD (*n* = 995)	*P* value
Age, years	56.8 ± 12.4	65.3 ± 10.8	<0.001
Male, *n* (%)	980 (54.1%)	710 (71.4%)	<0.001
BMI, kg/m^2^	25.4 ± 3.6	26.1 ± 3.7	<0.001
Hypertension, *n* (%)	820 (45.3%)	740 (74.4%)	<0.001
Diabetes mellitus, *n* (%)	240 (13.2%)	310 (31.2%)	<0.001
Current/ever smoker, *n* (%)	520 (28.7%)	410 (41.2%)	<0.001
Chronic kidney disease, *n* (%)	90 (5.0%)	120 (12.1%)	<0.001
Prior stroke/TIA, *n* (%)	70 (3.9%)	95 (9.5%)	<0.001
Lipid-lowering therapy (any), *n* (%)	330 (18.2%)	910 (91.5%)	<0.001
└ Statin, *n* (%)	300 (16.6%)	885 (88.9%)	<0.001
└ Ezetimibe, *n* (%)	25 (1.4%)	120 (12.1%)	<0.001
└ PCSK9 inhibitor, *n* (%)	2 (0.1%)	25 (2.5%)	<0.001
Folate/B-vit supplementation, *n* (%)	20 (1.1%)	60 (6.0%)	<0.001

Values are presented as mean ± SD or *n* (%), as appropriate. *P* values were calculated using Student's *t*-test or Wilcoxon rank-sum test for continuous variables, and *χ*² test or Fisher's exact test for categorical variables. Baseline laboratory values are summarized using the first available summer measurement for each biomarker (paired cohort).

Use of LLT was markedly more common among participants with CHD, consistent with secondary prevention practice; in contrast, LLT use in the non-CHD group likely reflected dyslipidemia management and/or primary prevention indications (Table 1). Importantly, the between-group patterns in seasonal changes were broadly preserved in prespecified analyses stratified by LLT status (Supplementary Table S1). In particular, triglyceride seasonal differences (Δ winter − summer) remained significantly divergent between CHD and non-CHD participants in both LLT and no-LLT strata, and HDL-C showed a similar direction of difference, reaching statistical significance among those receiving LLT (Supplementary Table S1).

### Within-Individual seasonal comparisons

**Figure 1:**
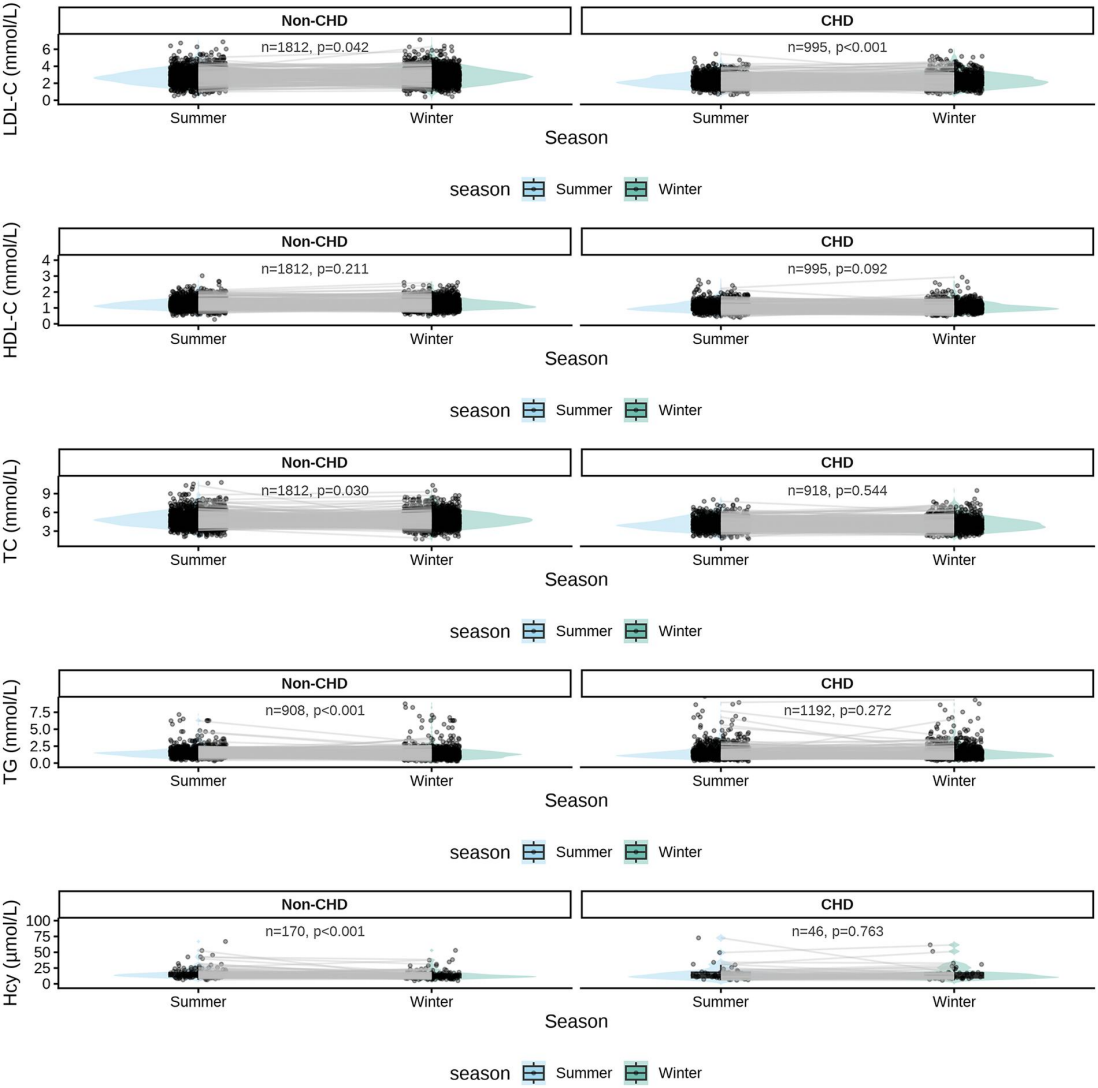
Paired seasonal distributions of lipid and homocysteine levels in individuals with and without coronary heart disease. Box-and-violin plots show the distributions of biomarker levels in summer and winter. Each dot represents an individual observation, and thin lines connect paired summer and winter measurements for a subset of individuals. Panels are stratified by biomarker (rows) and coronary heart disease (CHD) status (columns). *P* values indicate within-individual seasonal comparisons.

Within-group paired comparisons of summer and winter measurements are summarized in Table 2 and illustrated in Figure 1. Overall, seasonal differences were detectable but small in magnitude, with distinct patterns observed between non-CHD individuals and patients with CHD.

**Table 2:** Seasonal comparison of lipid profiles and homocysteine levels in individuals with and without coronary heart disease.

Biomarker	CHD status	*n* (Summer)	Summer, mean ± SD	*n* (Winter)	Winter, mean ± SD	Δ (Winter − Summer), mean ± SD	*P* value
LDL-C	Non-CHD	1,812	2.77 ± 0.89	1,812	2.96 ± 3.88	0.19 ± 3.89	0.042
LDL-C	CHD	995	2.30 ± 0.74	995	2.38 ± 0.81	0.08 ± 0.70	NA
HDL-C	Non-CHD	1,812	1.23 ± 0.31	1,812	1.24 ± 0.31	0.01 ± 0.25	0.211
HDL-C	CHD	995	1.08 ± 0.31	995	1.07 ± 0.30	−0.01 ± 0.24	0.092
TC	Non-CHD	1,812	4.94 ± 1.17	1,812	4.89 ± 1.12	−0.05 ± 1.06	0.030
TC	CHD	918	4.16 ± 1.01	918	4.18 ± 1.07	0.02 ± 0.93	0.544
TG	Non-CHD	908	1.60 ± 0.80	908	1.48 ± 0.96	−0.11 ± 1.02	NA
TG	CHD	1,192	1.55 ± 0.99	1,192	1.57 ± 1.07	0.03 ± 0.94	0.272
Hcy	Non-CHD	170	16.29 ± 8.12	170	13.54 ± 6.27	−2.75 ± 6.38	NA
Hcy	CHD	46	16.58 ± 11.89	46	16.11 ± 10.78	−0.47 ± 10.59	0.763

Values are presented as mean ± SD. Δ (Winter − Summer) represents the within-individual seasonal difference. *P* values were derived from paired *t*-tests or Wilcoxon signed-rank tests, as appropriate. Units: LDL-C, HDL-C, TC and TG are expressed in mmol/L; Hcy in μmol/L.

In the non-CHD group, LDL-C levels were modestly higher in winter compared with summer, while TC levels showed a slight winter decrease. These differences reached statistical significance but were small in absolute terms. Visual inspection of paired distributions demonstrated a subtle rightward shift of winter LDL-C values and a mild leftward shift of TC values, indicating consistent within-individual trends despite considerable overlap between seasons (Figure 1). Other lipid parameters, including HDL-C and TG, exhibited minimal seasonal variation. Hcy showed a numerically larger winter reduction in non-CHD individuals; however, variability was substantial across individuals.

In contrast, CHD patients displayed largely stable lipid and Hcy profiles across seasons. LDL-C exhibited only a minimal winter increase, and TC and TG remained essentially unchanged between summer and winter. HDL-C tended to be slightly lower in winter, although this difference did not reach conventional statistical significance. The paired plots demonstrated marked overlap between summer and winter distributions for most biomarkers, consistent with an attenuated seasonal response in the CHD population (Figure 1).

### Between-Group comparisons of seasonal differences

**Figure 2:**
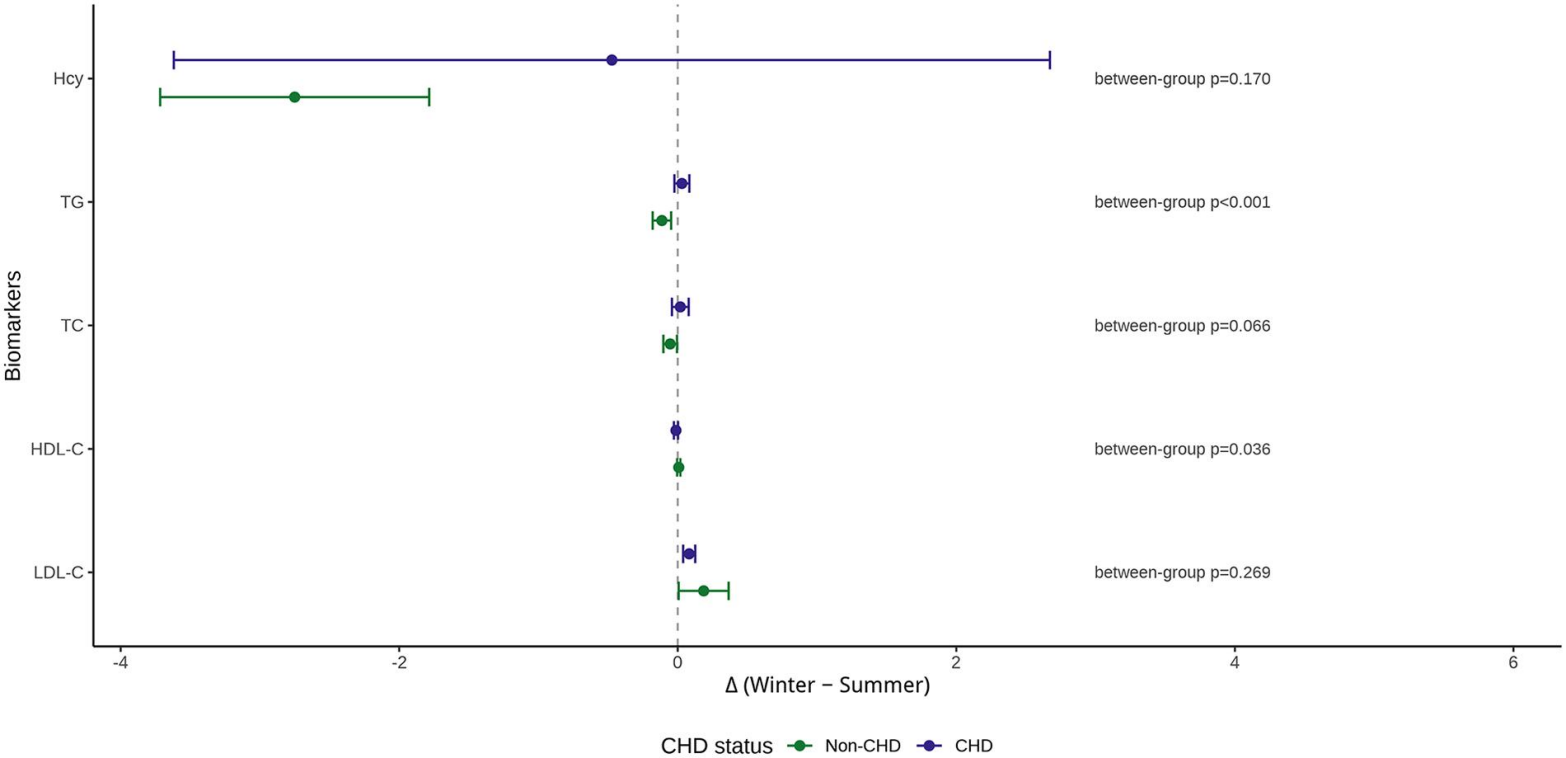
Seasonal differences (Δ winter − summer) in lipid and homocysteine levels by coronary heart disease status. Points represent mean seasonal differences (Δ Winter − Summer), and horizontal lines denote 95% confidence intervals. Biomarkers are shown on the *y*-axis, with positive values indicating higher levels in winter compared with summer. Results are stratified by CHD status. The vertical dashed line at zero indicates no seasonal difference.

Direct comparisons of seasonal differences (Δ Winter − Summer) between non-CHD and CHD groups are presented in Table 3 and summarized visually in Figure 2. Among all biomarkers examined, TG and HDL-C emerged as the primary sources of between-group heterogeneity.

**Table 3:** Between-group comparison of seasonal differences (Δ winter − summer) in lipid and homocysteine levels.

Biomarker	Non-CHD, mean ± SD	CHD, mean ± SD	*P* value
LDL-C	0.19 ± 3.89	0.08 ± 0.70	0.269
HDL-C	0.01 ± 0.25	−0.01 ± 0.24	0.036
TC	−0.05 ± 1.06	0.02 ± 0.93	0.066
TG	−0.11 ± 1.02	0.03 ± 0.94	<0.001
Hcy	−2.75 ± 6.38	−0.47 ± 10.59	0.170

Δ (Winter − Summer) indicates the seasonal change for each individual. *P* values were calculated using independent-sample *t*-tests or Wilcoxon rank-sum tests, as appropriate. Units: LDL-C, HDL-C, TC and TG are expressed in mmol/L; Hcy in μmol/L.

For TG, non-CHD individuals showed a tendency toward lower levels in winter, whereas CHD patients exhibited a slight winter increase, resulting in a highly significant between-group difference in Δ. This divergence was clearly depicted in the forest/dumbbell plot, where the confidence intervals for non-CHD and CHD TG estimates were positioned on opposite sides of the null reference line (Figure 2).

HDL-C also demonstrated a statistically significant between-group difference in Δ, characterized by a mild winter increase in non-CHD individuals and a mild winter decrease in CHD patients. Although the absolute changes were small, the opposite directional pattern contributed to statistical significance.

Between-group differences in Δ for LDL-C and TC were not statistically significant, with overlapping confidence intervals crossing zero. Hcy showed a larger numerical winter reduction in non-CHD individuals, but marked variability—particularly in the CHD group—precluded a statistically significant between-group difference.

### Integrated seasonal metabolic patterns

**Figure 3:**
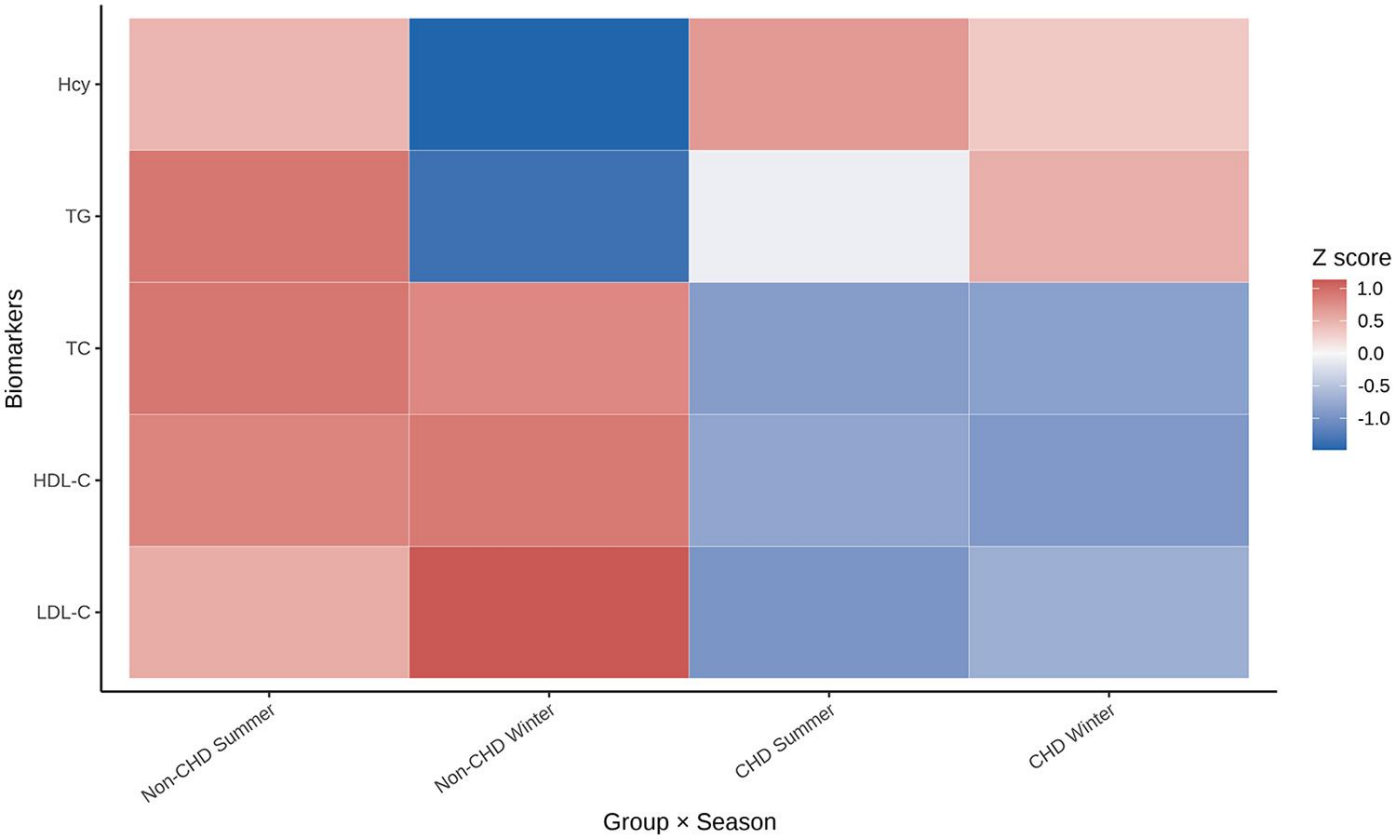
Metabolic signatures of lipid and homocysteine profiles across seasons and coronary heart disease status. Heatmaps display standardized mean levels (*Z* scores) of each biomarker across combinations of season and CHD status. *Z* scores were calculated within each biomarker to highlight relative seasonal patterns rather than absolute concentrations. Warmer colors indicate higher relative levels, and cooler colors indicate lower relative levels.

To provide a global overview of seasonal metabolic profiles, standardized mean biomarker levels were visualized using heatmaps (Figure 3). After normalization within each biomarker, non-CHD individuals exhibited relatively lower winter TG and Hcy levels compared with summer, whereas CHD patients showed less pronounced seasonal contrast, particularly for TG. LDL-C demonstrated a relative winter increase in non-CHD individuals but remained closer to neutral in CHD patients. These patterns were consistent with the paired and Δ-based analyses and highlighted systematic differences in seasonal response between clinical strata.

### Effect size and robustness analyses

**Figure 4:**
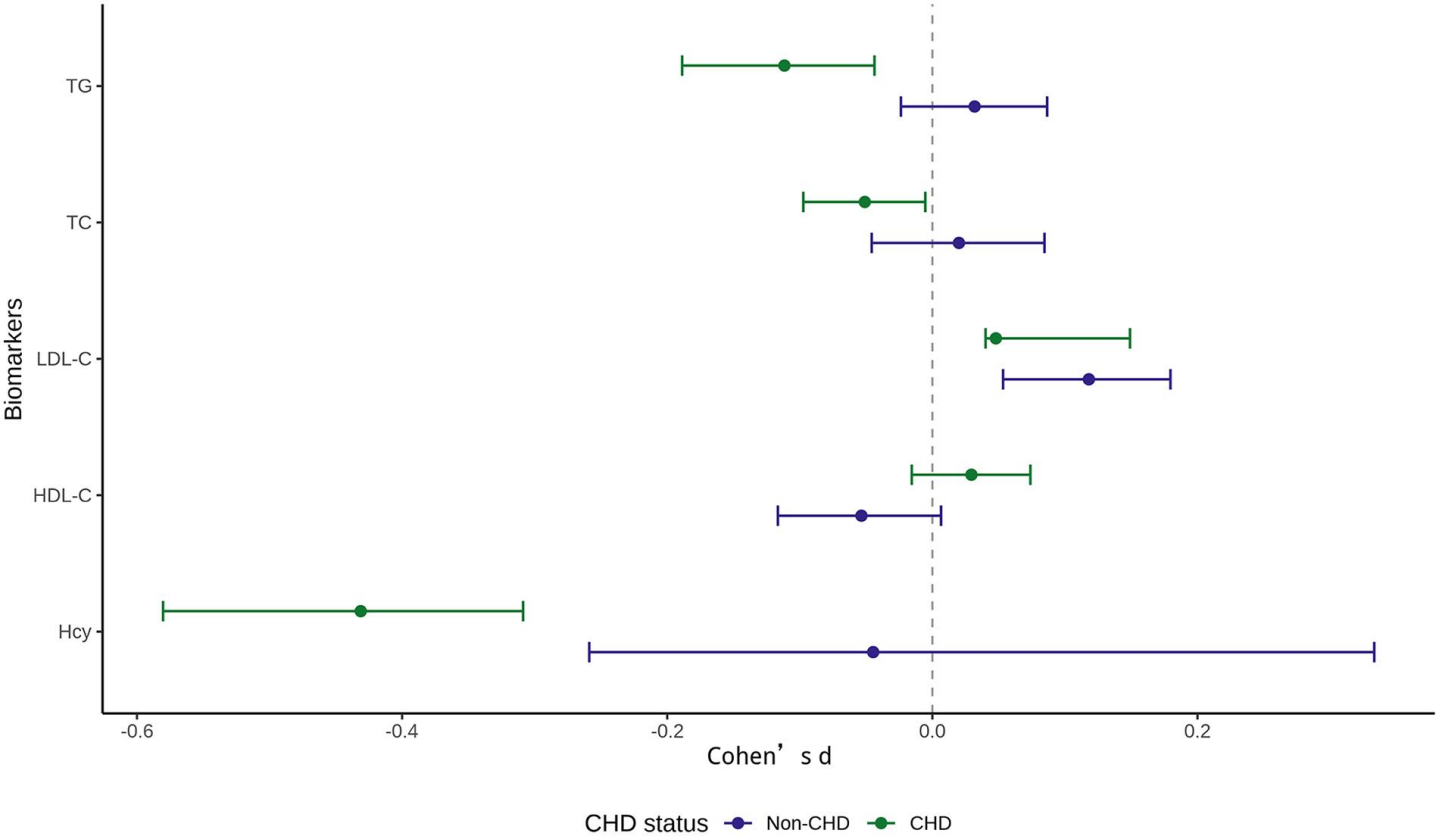
Effect size estimation of seasonal differences in lipid and homocysteine levels by coronary heart disease status. Points represent Cohen's d estimates for within-individual seasonal differences (winter vs summer), and horizontal lines denote 95% confidence intervals. Biomarkers are shown on the *y*-axis, with effect sizes stratified by coronary heart disease (CHD) status. Positive values indicate higher levels in winter than in summer, whereas negative values indicate lower levels in winter. The vertical dashed line at zero indicates no seasonal effect.

Effect size estimation using Cohen's d is shown in Figure 4. Across biomarkers and strata, most effect sizes were small and their confidence intervals frequently spanned zero, indicating that seasonal effects were generally modest. Notably, TG exhibited opposite effect directions between non-CHD and CHD groups, reinforcing the between-group findings and underscoring that directional heterogeneity, rather than large absolute changes, accounted for the observed statistical differences.

Sensitivity analyses using winsorized Δ values confirmed the robustness of the primary results (Supplementary Figure S1). The between-group difference in TG persisted after truncation of extreme values, and the overall pattern of small seasonal effects across other biomarkers remained unchanged. Examination of Δ distributions further illustrated the heterogeneity inherent to real-world data, with wider and more skewed distributions observed for TG and Hcy (Supplementary Figure S2).

## Discussion

In this real-world paired seasonal analysis from a cold-region clinical setting, we observed statistically detectable yet overall small seasonal shifts in lipid biomarkers and homocysteine, with a consistent pattern suggesting that Non-CHD individuals exhibited more apparent seasonal responsiveness whereas CHD patients showed a blunted or directionally altered response. This finding aligns with extensive epidemiologic evidence that cold seasons are associated with higher cardiovascular event rates and mortality, including winter peaks in acute myocardial infarction (AMI) incidence and death, as well as the broader temperature-attributable mortality burden predominantly driven by cold exposure (2, 9–11). Short-term temperature drops have been linked to increased MI risk in large population studies, supporting the biological plausibility that cold exposure may act as a trigger for acute coronary events (1). Collectively, these data reinforce the clinical relevance of seasonality as an environmental context in cardiovascular prevention and risk interpretation, especially in cold climates.

A key observation in our study was that LDL-C tended to increase slightly in winter, particularly detectable in the Non-CHD group (paired comparison), whereas CHD patients demonstrated smaller seasonal variability. Seasonal lipid variation has been repeatedly documented in population studies, with total cholesterol/LDL-C often higher in winter and lower in summer, although the magnitude and direction can differ by setting and population structure (4, 12–14). Mechanistically, multiple explanations have been proposed, including seasonal changes in plasma volume, physical activity, dietary composition, daylight exposure, and endocrine rhythms, which may jointly influence lipid concentrations and hepatic lipid metabolism (15). Importantly, the attenuated seasonal change in CHD patients in our cohort is clinically plausible because contemporary secondary prevention strategies—especially statin-based lipid lowering—stabilize LDL-C levels and reduce variability over time (6, 7). Hence, the “flatter” seasonal profile in CHD may reflect treatment effects and structured follow-up, whereas Non-CHD individuals may have greater lifestyle-driven fluctuations. Notably, the comparatively attenuated seasonal lipid variation observed among participants with CHD may, at least in part, reflect pharmacologic stabilization under contemporary secondary prevention, given the markedly higher prevalence of LLT in this group; meanwhile, a subset of non-CHD participants also received LLT for dyslipidemia and/or primary prevention in routine care. Accordingly, treatment status should be explicitly considered when interpreting between-group differences in seasonal lipid responses, and our LLT-stratified analyses provide complementary context for these comparisons.

The most robust between-group signal emerged from the discordant seasonal response of TG, with Non-CHD showing a winter decrease while CHD showed a slight winter increase, yielding a significant group difference in Δ (winter − summer). TG is particularly sensitive to short-term dietary patterns, physical activity, insulin resistance, and alcohol intake, all of which may exhibit seasonal variation, but can also be strongly modified by pharmacotherapy and comorbidity profiles in CHD populations (16–18). From a prevention perspective, TG is increasingly recognized as a marker of residual atherosclerotic risk even under statin therapy; outcome trials evaluating TG-targeted strategies have shaped current thinking. In REDUCE-IT, high-dose icosapent ethyl reduced major cardiovascular events among statin-treated patients with elevated TG (19), and in JELIS, eicosapentaenoic acid (EPA) reduced coronary events in a Japanese population receiving statins (20). Conversely, PROMINENT (pemafibrate) effectively lowered TG but did not reduce cardiovascular events, underscoring that TG lowering *per se* may not always translate into outcome benefit depending on drug class, mechanisms, and achieved lipid phenotype (21). Against this background, our finding that TG exhibits directionally different seasonal behavior between CHD and Non-CHD suggests that winter TG management might warrant more individualized attention in secondary prevention programs, especially in cold regions where lifestyle constraints and metabolic stressors may be amplified.

Another notable between-group difference involved HDL-C, where the CHD group showed a small winter decrease while Non-CHD remained essentially stable. HDL-C is influenced by activity level, adiposity, smoking, insulin sensitivity, and inflammatory state—factors that may worsen in winter, particularly in populations with established cardiometabolic disease (17, 18). While HDL-C is no longer viewed as a straightforward causal target, its seasonal decline in CHD may reflect a winter shift toward a less favorable metabolic milieu and can serve as a clinically interpretable signal prompting reinforcement of physical activity and weight control strategies during cold months. In addition, climate parameters beyond temperature—such as humidity, daylight length, and atmospheric conditions—have been associated with lipid seasonality in longitudinal analyses, indicating that “season” is a composite exposure rather than a single factor (14, 15).

Hcy displayed a distinct pattern: a larger winter decrease in Non-CHD but not in CHD, although between-group Δ did not reach statistical significance. Elevated Hcy has been linked to vascular risk in observational studies, but randomized trials of B-vitamin therapy to lower Hcy have generally failed to demonstrate cardiovascular event reduction in secondary prevention settings, including after MI (NORVIT) and after ischemic stroke (VISP) (22–24). These trial data caution against over-interpreting Hcy shifts as causal; nonetheless, seasonal Hcy variability may still matter for risk stratification, laboratory interpretation, and hypothesis generation regarding nutritional intake (folate/B vitamins), renal function, medication use, and inflammatory status—factors likely to differ systematically between Non-CHD and CHD clinical populations. Therefore, we recommend that future work incorporate medication data (e.g., folate/B vitamins, statins), renal indices, and dietary proxies to clarify whether observed seasonal Hcy changes reflect true biology or compositional differences in who gets tested in each season.

From a pathobiological perspective, our clinical signals are directionally compatible with emerging mechanistic evidence that cold exposure can influence atherosclerosis progression and plaque stability through immunometabolic pathways. Experimental and translational literature has implicated cold-induced thermogenic programs, adipose-derived mediators, and macrophage metabolic stress in modulating atherogenesis and inflammatory tone (8, 25, 26). In particular, impaired clearance of apoptotic cells (efferocytosis) is recognized as a contributor to necrotic core expansion and plaque vulnerability; “find-me” signaling and phagocyte recruitment are critical components of effective efferocytosis biology (27–29). Although our dataset does not measure inflammation or plaque characteristics directly, the observed winter shift toward a less favorable lipid profile in CHD may be consistent with a broader cold-season pro-atherogenic milieu suggested by mechanistic studies. This provides a rationale to integrate metabolic biomarkers with inflammatory markers (e.g., hsCRP), medication adherence data, and clinical outcomes in future studies.

Clinically, these findings support a pragmatic approach: in cold regions, clinicians may consider winter-focused reinforcement of lifestyle counseling and lipid management, particularly for CHD patients where TG and HDL-C showed directionally less favorable seasonal behavior and where residual risk remains relevant even under statin therapy. For Non-CHD individuals, seasonal shifts—though small—could influence screening interpretation and risk communication; clinicians should avoid overreacting to single measurements without considering seasonal context. Overall, our study adds real-world paired evidence that seasonal metabolism is heterogeneous across cardiovascular risk strata, and it motivates multi-season, multi-marker integrated management strategies in cold-climate populations.

### Study limitations

Several methodological considerations are important when interpreting our findings. First, effect sizes were generally small, highlighting that statistical significance—especially in large real-world samples—does not necessarily imply clinical relevance; reporting standardized effect sizes alongside *p*-values helps contextualize “detectable but mild” seasonal changes. Second, real-world laboratory datasets often show heteroscedasticity and long-tailed distributions (notably for TG and Hcy), making robust approaches (e.g., winsorization, sensitivity analyses) valuable to ensure conclusions are not driven by outliers; our robustness checks supported the stability of the main TG signal. Third, the retrospective single-center design limits causal inference and is vulnerable to unmeasured confounding (fasting status, diet, exercise, socioeconomic factors, heating exposure, and medication adherence). Finally, reporting should follow established observational study guidance (STROBE), and future work would benefit from prespecified analytic plans, multiple-comparison control (e.g., FDR), and linkage to hard outcomes (AMI, hospitalization, death) to translate biomarker seasonality into actionable risk estimates (30).

## Conclusions

This real-world paired study demonstrates that seasonal variation in lipid profiles and homocysteine is statistically detectable but generally modest, with clear heterogeneity between individuals with and without coronary heart disease. Non-CHD individuals showed greater seasonal sensitivity, whereas CHD patients exhibited attenuated or directionally different responses, particularly for triglycerides and HDL-C. Although effect sizes were small, these consistent directional differences highlight the importance of considering seasonal context when interpreting lipid measurements and optimizing individualized cardiovascular risk management in cold-climate regions.

## Data Availability

The original contributions presented in the study are included in the article/Supplementary Material, further inquiries can be directed to the corresponding author.
