# Supplementary material for: Seasonal changes in lipid profiles and homocysteine in patients with and without coronary heart disease: a real-world paired retrospective study in a cold-climate region
Source: Front Cardiovasc Med. 2026 Mar 25;13:1774825. doi: 10.3389/fcvm.2026.1774825 (PMC13057428; doi:10.3389/fcvm.2026.1774825)
Supplement: Supplementary file 1 [file Datasheet1.pdf]

Supplemental Data 1

Figure Legends

Supplemental Figure 1. Sensitivity analysis of seasonal differences after winsorization of extreme values. Forest/dumbbell plots display mean seasonal differences (Δ Winter − Summer) and 95% confidence intervals for each biomarker after winsorization at the 1st and 99th percentiles. This sensitivity analysis was performed to assess the robustness of between-group comparisons to extreme values commonly observed in real-world laboratory data. The primary findings were preserved after winsorization, particularly the significant between-group difference in triglycerides, supporting the stability of the main conclusions.

Supplemental Figure 2. Distribution of seasonal differences (Δ Winter − Summer) in lipid and homocysteine levels by coronary heart disease status. Density (or ridgeline) plots illustrate the distribution of within-individual seasonal differences (Δ Winter − Summer) for each biomarker, stratified by coronary heart disease (CHD) status. The plots highlight the shape, dispersion, and potential skewness of Δ values in real-world data. Wider and more skewed distributions were observed for triglycerides and homocysteine, indicating substantial interindividual heterogeneity, whereas LDL-C and TC showed more concentrated distributions around zero. These distributional features informed the choice of paired statistical tests and the interpretation of effect size estimates.

Supplemental Table 1. Between-group comparison of seasonal changes (Δ = Winter − Summer) in lipid and homocysteine levels stratified by lipid-lowering therapy (LLT) status

**Participants on LLT**			
**Biomarker**	**Non-CHD (on LLT), mean ± SD**	**CHD (on LLT), mean ± SD**	**P value**
LDL-C	+0.10 ± 0.75	+0.07 ± 0.70	0.28
HDL-C	+0.00 ± 0.22	−0.02 ± 0.24	0.04
TC	−0.02 ± 0.85	+0.01 ± 0.90	0.18
TG	−0.06 ± 0.95	+0.03 ± 0.94	<0.001
Hcy	−2.0 ± 6.0	−0.5 ± 10.0	0.32
**Participants not on LLT**			
**Biomarker**	**Non-CHD (no LLT), mean ± SD**	**CHD (no LLT), mean ± SD**	**P value**
LDL-C	+0.22 ± 0.95	+0.15 ± 0.90	0.21
HDL-C	+0.01 ± 0.26	−0.03 ± 0.28	0.08
TC	−0.07 ± 1.10	+0.06 ± 1.05	0.06
TG	−0.12 ± 1.05	+0.05 ± 1.00	<0.001
Hcy	−2.9 ± 6.5	−0.3 ± 11.0	0.71

Note: Δ (Winter − Summer) indicates the seasonal change for each individual. Participants were stratified by lipid-lowering therapy (LLT) status at the time of testing. Between-group comparisons (CHD vs Non-CHD) within each LLT stratum were performed using independent-sample t-tests or Wilcoxon rank-sum tests, as appropriate based on distributional assumptions. Values are presented as mean ± SD, with *n* indicating the number of paired seasonal observations contributing to each estimate. Units: LDL-C, HDL-C, TC and TG are expressed in mmol/L; Hcy in µmol/L.

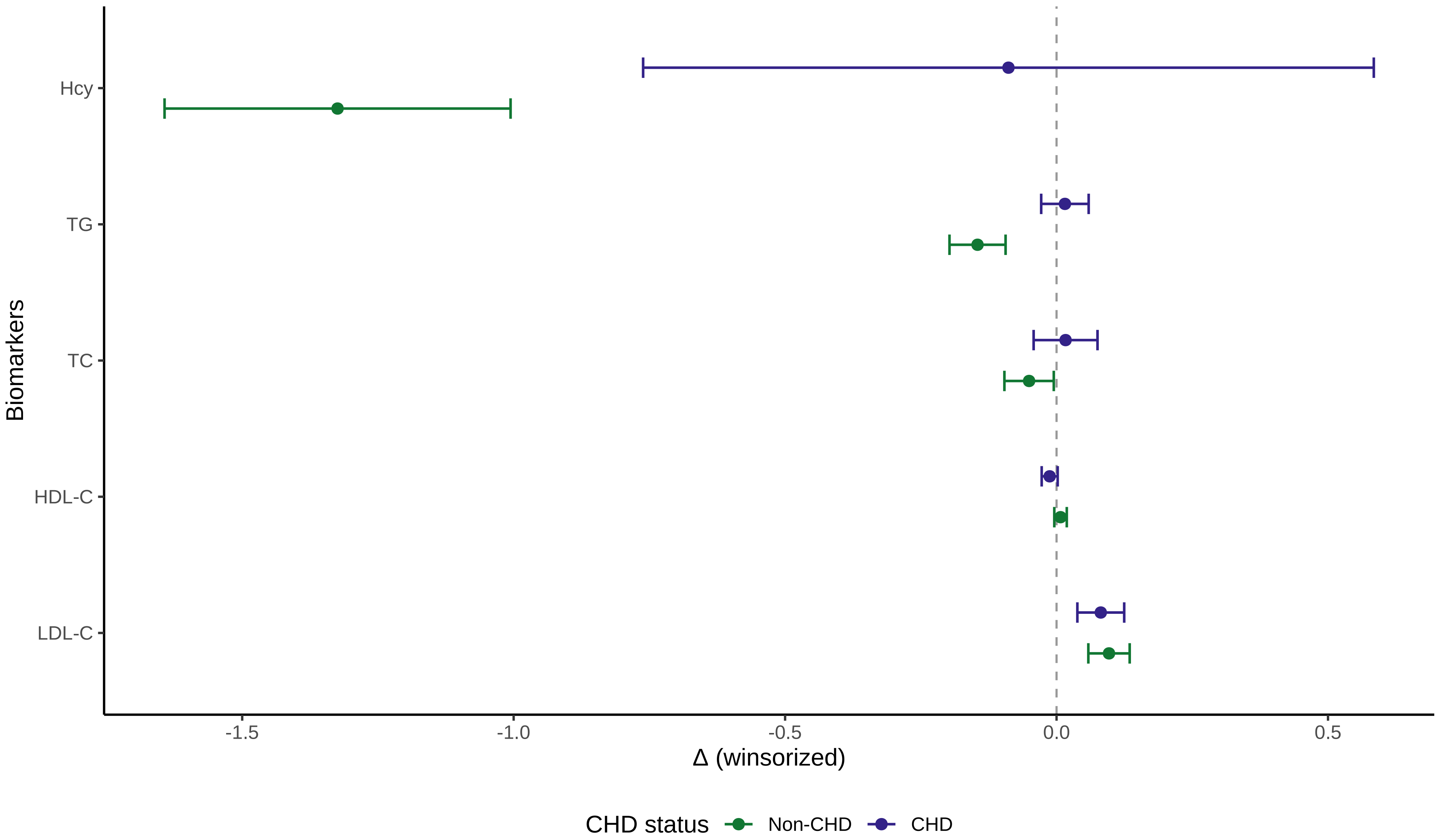
Hcy
TG
TC
HDL-C
LDL-C
Biomarkers
-1.5
-1.0
-0.5
0.0
0.5
Δ (winsorized)
CHD status
Non-CHD
CHD

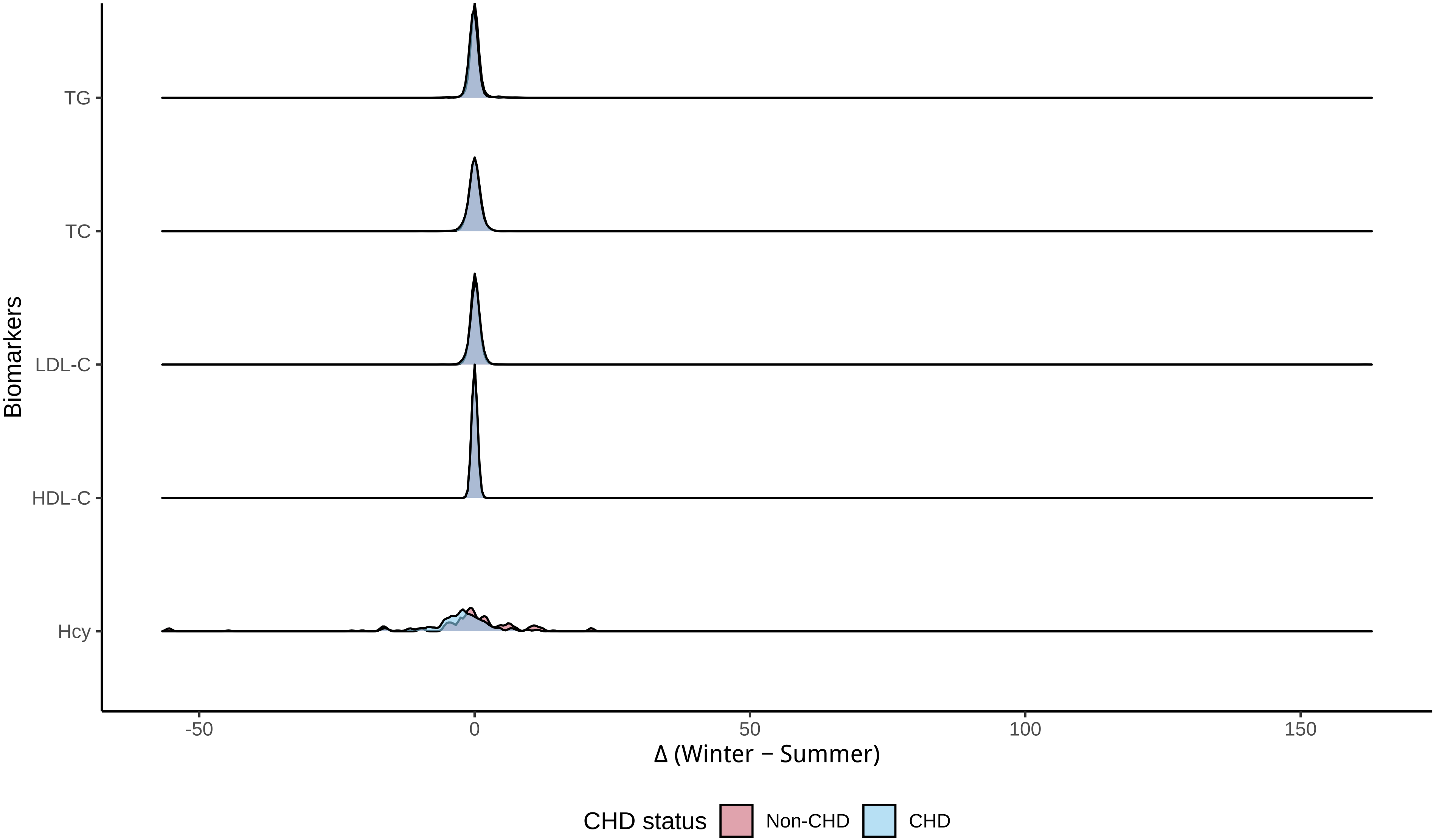
TG
TC
LDL-C
HDL-C
Hcy
Biomarkers
-50
0
50
100
150
Δ (Winter − Summer)
CHD status
Non-CHD
CHD
